# Characterization of variants in the promoter of BZLF1 gene of EBV in nonmalignant EBV-associated diseases in Chinese children

**DOI:** 10.1186/1743-422X-7-92

**Published:** 2010-05-10

**Authors:** Yingkang Jin, Zhengde Xie, Gen Lu, Shuang Yang, Kunling Shen

**Affiliations:** 1 Department of Infection, Beijing Children's Hospital, The Capital Medical University, Beijing 100045, China; 2 Department of Virology, Beijing Children's Hospital, The Capital Medical University, Beijing 100045, China; 3 Department of Hematology, Beijing Children's Hospital, The Capital Medical University, Beijing 100045, China

## Abstract

**Background:**

Diseases associated with Epstein-Barr virus (EBV) infections, such as infectious mononucleosis (IM), EBV-associated hemophagocytic lymphohistiocytosis (EBV-HLH) and chronic active EBV infection (CAEBV) are not rare in Chinese children. The association of type 1 or type 2 EBV and variants of the EBV BZLF1 promoter zone (Zp) with these diseases is unclear.

**Results:**

The objective of this study was to investigate the relationship between EBV genotypes (Zp variants and EBV type 1 and 2) and the clinical phenotypes of EBV-associated diseases in Chinese children. The Zp region was directly sequenced in 206 EBV-positive DNA samples from the blood of patients with IM, EBV-HLH, CAEBV, and healthy controls. Type 1 or type 2 EBV was examined by PCR for EBNA2 and EBNA3C subtypes. Four polymorphic Zp variants were identified: Zp-P, Zp-V3, Zp-P4 and Zp-V1, a new variant. The Zp-V3 variant was significantly associated with CAEBV (*P *≤ 0.01). The frequency of co-infection with Zp variants was higher in patients with CAEBV and EBV-HLH, compared with IM and healthy controls, mostly as Zp-P+V3 co-infection. Type 1 EBV was predominant in all categories (81.3-95%) and there was no significant difference in the frequency of the EBV types 1 and 2 in different categories (*P *> 0.05).

**Conclusions:**

Type 1 EBV and BZLF1 Zp-P of EBV were the predominant genotypes in nonmalignant EBV associated diseases in Chinese children and Zp-V3 variant may correlates with the developing of severe EBV infection diseases, such as CAEBV and EBV-HLH.

## Background

*Epstein-Barr virus *(EBV) is a member of the *Lymphocryptovirus *genus, *Gammaherpesvirinae *subfamily of the *Herpesviridae *family of viruses. This virus is associated with a wide variety of diseases, both benign and malignant, which ubiquitously infect humans and persist for the lifetime of the individual. During its life cycle, EBV has latent and productive (lytic) phases. The latent phase maintains the virus long-term in its host and can lead to the productive phase where virus is reactivated and produced allowing it to be transmitted. During the two phases, EBV expresses a set of viral gene products in its life cycle and some of these genes were proved to possess the potential to cause changes in the interactions between the virus and the host's immune system [1,2].

The biology and pathogenesis of EBV has been the focus of many studies but the clinical management of the disease is poorly understood. Whether certain EBV genotypes are involved in the pathogenesis of specific EBV-related diseases has been the subject of investigation in recent years. Several viral variants can be distinguished according to polymorphisms in EBV genes, such as EBV nuclear antigen (EBNA) and BZLF1, a potent regulator of the switch from latency to lytic phases encoded by the EBV *Bam*HI fragment Z. EBV genotypes can be categorized as type 1 or type 2 on the basis of marked allelic polymorphisms within the EBNA2, 3A, 3B, and 3C genes [3,4]. Both EBV types have been detected in immunocompromised and immunocompetent hosts but type 1 EBV is predominant in Asian nasopharyngeal carcinoma and has a greater potential to transform B lymphocytes than EBV type 2. Type 2 EBV, on the other hand, enters the lytic cycle more readily than type 1 EBV [5-7]. Sequence diversity of the BZLF1 gene promoter zone (Zp) (from -221 to +12, with respect to the transcription start site of BZLF1) have also been identified and variants are differentially distributed among malignant and non-malignant cells [8,9].

Childhood EBV infection is typically asymptomatic but can also induce three types of non-malignant disorders, including infectious mononucleosis (IM), EBV-associated hemophagocytic lymphohistiocytosis (EBV-HLH) and chronic active EBV infection (CAEBV). Certain linkages exist between these diseases where IM, usually a benign self-limiting disease, can develop to EBV-HLH and CAEBV in some patients. Likewise, EBV-HLH progresses very rapidly and becomes a life-threatening disease without immunosuppressive therapy, which occurs during the process of CAEBV sometimes or in association with fulminant IM[10-12]. CAEBV is characterized by chronic or recurrent IM-like symptoms persisting over a long period of time and has a high likelihood of developing into EBV related malignant diseases, such as T/NK cell lymphomas, with a high fatality rate [13-15]. Thus, this study aimed to investigate the association of BZLF1 Zp variants and type 1 and type 2 EBV and to explore the relationship between these EBV genotypes and clinical phenotypes of EBV-associated diseases in Chinese children.

In this study, EBV DNA from blood samples of 206 patients with IM, EBV-HLH, CAEBV, and healthy controls was examined by PCR for EBNA2 and EBNA3C subtypes (EBV type 1 and type 2) and Zp variants. This case-control study is the first investigation to explore the association between EBV subtypes and BZLF1-Zp variants and EBV infection in the China children population.

## Results

### Definition of type 1 or/and type 2 EBV in patients with EBV infection

The frequency of type 1 or type 2 EBV infection was determined for all samples (Table 1). Collectively, type 1 EBV was present in 190 of 206 samples (92.2%) and type 2 EBV was found in 12 samples (5.8%). Among all patients, there was no significant difference (*P *> 0.05) in the frequency of the EBV type 1 and type 2 between categories. The remaining four cases (1.9%) displayed co-infection with both type 1 and type 2 EBV and were all from the CAEBV group.

**Table 1:** The frequency of EBV types 1 and 2 in each EBV-related disease group

Groups	EBV subtypes (n/N)		
	
	Type 1	Type 2	Type 1 + Type 2
IM (n = 88)	94.3 (83/88)	5.7 (5/88)	0
HLH (n = 46)	93.4 (43/46)	6.6 (3/46)	0
CAEBV (n = 32)	81.3 (26/32)	6.2 (2/32)	12.5 (4/32)
Controls (n = 40)	95.0 (38/40)	5.0 (2/40)	0
Total (n = 206)	92.2 (190/206)	5.8 (12/206)	1.9(4/206)

### Zp variants in EBV infected children

**Figure 1:**
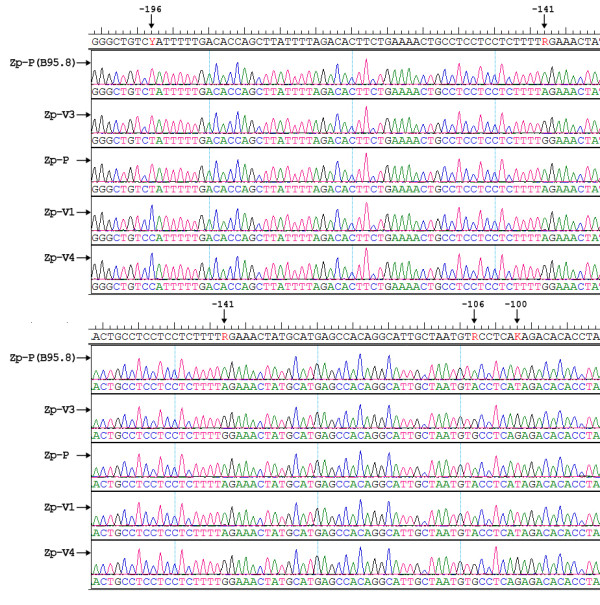
**DNA sequences obtained for four EBV BZLF1 gene promoter zone (Zp) variants compared with the B95.8 prototype sequence**. Positions relative to the transcription start site are indicated.

Sequence differences identified within the major regulatory Zp domains (nucleotides -211 to +12) of EBV infected individuals can be grouped into four variant forms (Figure 1). Zp-P group sequences are identical to the EBV prototype strain, B95.8. Zp-V3 and Zp-V4 variants have been previously described by Gutierrez et al. [8]. Zp-V3 group sequences differ from Zp-P at three positions: -100 (T→G), -106 (A→G), and -141 (A→G); while Zp-4 sequences are characterized by same three substitutions of the Zp-V3 variant in addition to a T to C substitution at position -196. A new Zp variant was identified and named Zp-V1 and differs from Zp-P by a single substitution at position -196 (T→C). As shown in Table 2, The distribution of Zp subtypes involved all co-existence variants-IM included P(n = 61), V3(n = 3), V1(n = 4), V4(n = 6), P+V1(n = 6), P+V3(n = 2), P+V4(n = 5); EBV-HLH included P(n = 23), V3(n = 8), V4(n = 1), P+V3(n = 12), P+V4(n = 2); CAEBV included P(n = 6), V3(n = 14), P+V3(n = 12); controls included P(n = 29), V3 (n = 1), V1(n = 3), V4(n = 4), P+V1(n = 1), P+V4(n = 2). We found that Zp-P variant was the dominant genotype found in all infection categories, except a relatively rare disease-CAEBV, indicating that it was the primary variant of EBV circulating in China. The Zp-V3 variant was the dominant genotype in CAEBV cases (*P *≤ 0.01) and relatively high in EBV-HLH cases. The Zp-V1 variant, however, was only found in IM and control cases, while the V4 variant was not detected in any CAEBV cases.

**Table 2:** The frequency of EBV BZLF1 gene promoter zone (Zp) variants in each EBV-related disease group.^#^

Zp-variant %(n/N)	IM (n = 88)	HLH (n = 46)	CAEBV (n = 32)	Controls (n = 40)
P	84.1 (74/88)	80.4 (37/46)	56.3 (18/32)^△^	85.0 (34/40)
V3	5.7 (5/88)	43.5 (20/46)^△^	81.3 (26/32)^△^	2.5 (1/40)
V4	12.5 (11/88)	6.5 (3/46)	0	15.0 (6/40)
V1	11.4 (10/88)	0	0	10.0 (4/40)

^#^The distribution of Zp subtypes involved all co-existence variants-IM included P(n = 61), V3(n = 3), V1(n = 4), V4(n = 6), P+V1(n = 6), P+V3(n = 2), P+V4(n = 5); EBV-HLH included P(n = 23), V3(n = 8), V4(n = 1), P+V3(n = 12), P+V4(n = 2); CAEBV included P(n = 6), V3(n = 14), P+V3(n = 12); controls included P(n = 29), V3 (n = 1), V1(n = 3), V4(n = 4), P+V1(n = 1), P+V4(n = 2)

^△^(P ≤ 0.01) vs other groups

### Co-existence of Zp variants and EBV subtypes

**Figure 2:**
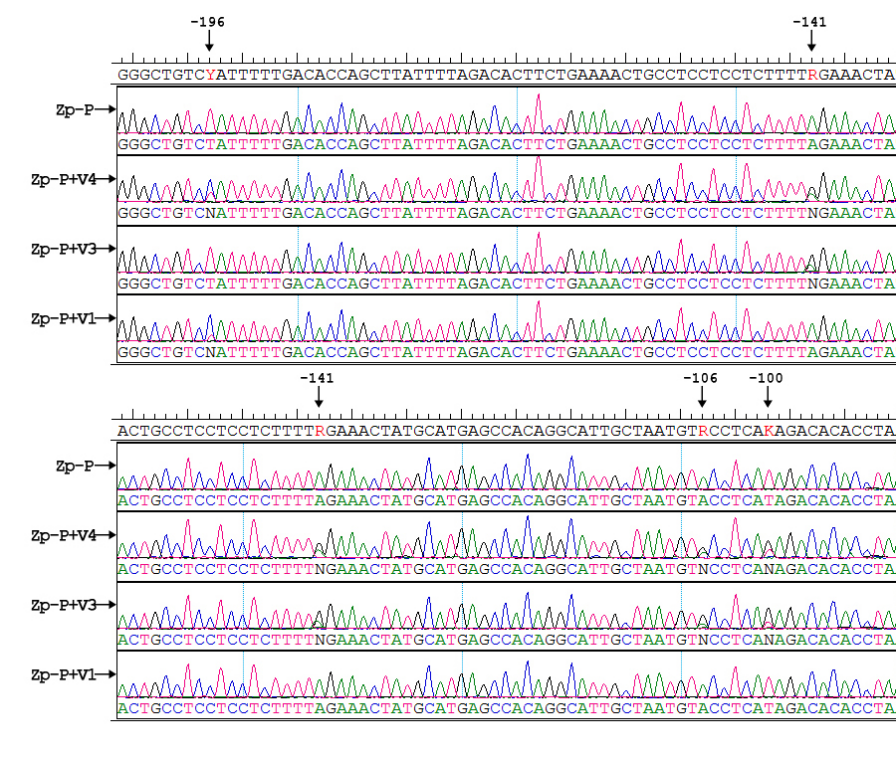
**DNA sequences obtained for co-exisence of EBV BZLF1 gene promoter zone (Zp) variants compared with the B95.8 prototype sequence**. Positions relative to the transcription start site are indicated.

As shown in Table 3, the incidence of co-existence of Zp variants (Figure 2) in HLH (30.4%) and CAEBV (37.5%) cases was higher than for both IM (14.8%) and control cases (7.5%). Interestingly, the Zp-P variant was present in every co-existence case that harbored two Zp variants. Zp-P+V1 variants were only detected in IM and control categories, whereas Zp-P+V3 variants were predominant in CAEBV and HLH samples. The four type 1+2 EBV co-infection cases detected in the CAEBV group all contained Zp-P+V3 variants.

**Table 3:** The co-existence of EBV BZLF1 gene promoter zone (Zp) variants and EBV subtypes in each EBV-related disease study group.

Co-infection %(n/N)	IM (n = 88)	HLH (n = 46)	CAEBV (n = 32)	Controls (n = 40)
Zp-P+V1	6.8 (6/88)	0	0	2.5(1/40)
Zp-P+V4	5.7 (5/88)	4.3 (2/46)	0	5.0 (2/40)
Zp-P+V3	2.3 (2/88)	26.1(12/46)	37.5(12/32)	0
Total	14.8 (13/88)	30.4(14/46)	37.5(12/32)	7.5 (3/40)

* Four patients detected in CAEBV with both type 1 and type 2 co-infections were also presented with Zp-P and Zp-V3 co-exsistences.

## Discussion

This case-control study is the first investigation to explore the association between EBV subtypes and BZLF1-Zp variants and EBV infection in the China children population. In this study, statistical analysis determined that differences in the distribution of Zp variants were significant in the four patient categories. The frequency of the Zp-V3 variant in the CAEBV group was statistically higher than for other categories (*P *≤ 0.01), while Zp-P was predominant in all categories except CAEBV. This suggests that the Zp-P variant EBV was the most common variant found in China and that infection by Zp-V3 is strongly correlated to CAEBV. The Zp-V3 variant is significantly associated with malignancy in both immunocompetent and immunocompromised patients [8,9] and the higher frequency of the Zp-V3 variant in CAEBV patients observed in the current study suggests that CAEBV is more likely an entity of pre-malignancy. Similarly, the Zp-V4 variant was also identified in this study and was most associated with IM and healthy control cases. Zp-V1 was identified as a novel variant and was detected in 10 IM and health control cases but not in CAEBV and EBV-HLH. The absence or low level of ZP-V1 and Zp-V4 in CAEBV and HLH reflects a less severe pathogenesis than for the Zp-V3 variant which may enhance the tumorigenicity of EBV.

A novel Zp variant that differed from Zp-P by one substitution at position -100 (T→G) was detect in this study in only one patient with EBV-HLH. Due to the infrequent isolation of this variant, we did not include this data in correlations with disease. Previously described Zp variants, Zp-PV, Zp-V1-104, Zp-V1-105 or Zp-V1-119 [9,16] were not detected in any patient samples. Although it may be chance that these isolates were not detected, specific ethnic groups and geographical restrictions are likely to contribute to the narrow distribution of variants observed in the current study. The detection of different new variants suggests that the accumulation of viral mutations may contribute to the variations observed within the host during virus persistence.

Similar to other studies that reported that type 1 EBV was predominant in Asian nasopharyngeal carcinoma (86.5-96%) [17,18], the current study also found that type 1 EBV was predominant in all four categories (81.3-95%). Also in agreement to these studies, type 2 EBV infection was rarely detected (4-13.5%). These findings suggest that the diagnosis of EBV types 1 and 2 in patients is not likely to be useful for predicting susceptibility to EBV-related diseases in Chinese children. Although patients with Zp-V4 or Zp-V1 variants were always type 1 EBV carriers, this study did not confirm that Zp variants segregated by EBV type due to the extremely lower frequency of type 2 EBV in the Chinese study population. Gutierrez et al. had previously shown that the Zp-V3 variant was exclusively associated with type 2 EBV infection; however, the current study found that variant Zp-V3 co-existed with both EBV types. Geographic regions, sample sizes or various diseases are like to result in these differences.

The prevalence of co-existence EBV Zp variants within the four categories studied ranged from 14.6 to 37.5%. The majority of these co-existence viruses occurred in patients with CAEBV and EBV-HLH and always was found associated with Zp-P and not other variants. It is likely that the majority of people are first infected with a more prevalent variant like Zp-P, the predominant variant found in this study, but does not rule out the possibility that new point mutations are likely to be arised during EBV replication of in its hosts from pre-existing variant. In this way, the balance of one pre-existing virus variant which could be controlled by its host, may be disturbed by a specific new variant. Thus, virus replication, tropism, or immune evasion in its hosts could be greatly enhanced after acquiring this new variant. As the Zp-V3 variant was associated with severe diseases in this study, the Zp-V3 type point mutations derived from Zp-P are likely to be associated with a more invasive capacity than Zp-V1 or Zp-V4 variants. Taken together, superinfection by multiple strains of EBV, especially the presence of the Zp-V3 variant, may be a contributing factor in the development of severe EBV infections in children. Thus, these findings may give some prospect to explore the differential distribution of Zp variants in susceptible populations and their association with severe or even fatal EBV diseases. A close dynamic follow-up on patients carrying EBV from an early stage of infection may help us understand how the host immune response allows such mutations to occur.

Just how an individual acquires such mixtures of Zp variants is unknown. This could occur by simultaneous acquisition or by the serial accumulation from exposure to different variant carriers. It seems implausible that such co-infections can be co-acquired from a carrier who was shedding multiple variants in saliva, because it is unclear how the source can accumulate multiple infections before transmitting those orally shedding multiple EBV variants to the next. As infection by EBV with the Zp-P variant was a prerequisite for co-existence in this study, it is possible that an individual is more likely to acquire a prevalent variant, such as Zp-P, at first exposure to the virus, and then the host immunity to this variant is developed. However, part of hosts may fall short in resisting another different variant the next time. It is more likely that some instances of co-existence are the result of serial acquisition over time from independent sources. The host could gain multiple variants dynamically in the light of this hypothesis, but this cannot explain why co-infections harbor no more than two variants. It is plausible that the co-existence variants observed in this study may be new variants that were generated from point mutations of a pre-existing variant. However, the complex relationship between EBV variants and the host requires further investigation of other EBV-related diseases, various ethnic groups, different tissues or a study including a larger sample size.

## Conclusion

In conclusion, this study described the EBV genotype profiles found in children with different EBV-related diseases. The most prevalent EBV genotypes found in Chinese children were type 1 EBV and the BZLF1 Zp-P variant. The results show that patients with the Zp-V3 variant may have a higher risk of developing severe EBV-associated diseases.

## Materials and methods

### Study subjects

A total of 206 whole blood samples were obtained from inpatients at Beijing Children's Hospital from 2006-2008 with an age range from 13 months to 12 years old. Blood was collected from 40 healthy control cases, 88 individuals diagnosed with IM, 46 with EBV-HLH and 32 with CAEBV based on their respective diagnostic criteria in previously literatures [19,11,20]. Genomic DNA was obtained from whole blood samples using a whole blood genomic DNA isolation kit (Tiangen, China). All the subjects were confirmed EBV-positive by conventional PCR or real-time PCR on DNA isolated from the peripheral blood leukocytes (PBL). Patients were categorized as having IM, EBV-HLH and CAEBV based on meeting the respective diagnostic criteria. The healthy control cases were enrolled on the basis of EBV-specific antibodies present in their blood detected by serologic tests for capsid antigens (CA) (Biochip; Euroimmun, Germany) but did not exhibit any clinical manifestation of EBV-related diseases.

All individuals in the present study are of Han descents and provided informed written consent to perform the study. All procedures were approved by the Committee of Human Studies at the Beijing Children's Hospital affiliated with the Capital University of Medical Sciences.

### EBNA2 and EBNA3C subtyping to define of type 1 and type 2 EBV

The determination of EBV type 1/type 2 infections was performed using a standard PCR assay across type-specific regions of EBNA2 and EBNA3C gene using primers for EBNA2: 5'-AGGCTGCCCACCCTGAGGAT-3' and 5'-GCCACCTGGCAGCCCTAAAG-3' and primers for EBNA3C: 5'-AGAAGGGGAGCGTGTGTTGT-3' and 5'-GGCTCGTTTTTGACGTCGGC-3', as previously described [21,22]. The reaction mixture (50 μL) was pre-incubated at 95°C for 5 min prior to thermocycling for 35 cycles at 95°C for 45 s, 56°C for 45 s, 72°C for 1 min followed by extension at 72°C for 10 min. PCR products were analyzed by electrophoresis migration in 2% agarose gel. PCR products amplified by EBNA2 primers yield a product of 168 base pairs (bp) for type 1 EBV and 184 bp for type 2 EBV. PCR products amplified with EBNA3C primers yield a product of 153 bp for type 1 EBV and 246 bp for type 2 EBV.

### BZLF1 Zp sequence analysis

The Zp genotype was investigated based on the sequence diversity between positions -211 to +12 (with reference to the transcription start site) of the BZLF1 promoter zone (Zp) by a single step PCR. Oligonucleotide primers (5'-AGCATGCCATGCATATTTC-3' and 5'-TTGGCAAGGTGCAATGTTT-3') were used to amplify genomic DNA extracted from PBL samples by PCR, as previously described before [8]. The reaction mixture (50 μL) was incubated at the thermocycling conditions of 5 min at 95°C, 35 cycles of 45 s at 95°C, 45 s at 57°C and 60 s at 72°C, followed by a final extension cycle of 10 min at 72°C. PCR products were separated by 2% agarose gel electrophoresis and purified. The products were used for direct sequencing with the same primers used for amplification. The EBV-positive cell line, B95.8, was used as a positive control to confirm or identify Zp-variants.

### Statistical analysis

Statistical analysis was performed using the SPSS (Version 11.0; SPSS, Chicago, USA) software package. The association of each polymorphic Zp with type 1 and 2 EBV and the distribution of the variants within different categories were analyzed using the chi-square analysis. All statistical tests were two-sided and *P*-values less than 0.05 were considered statistically significant.

## List of abbreviations

CA: capsid antigens; CAEBV: chronic active EBV infection; EBV: Epstein-Barr virus; EBNA: EBV nuclear antigen; EBV-HLH: EBV-associated hemophagocytic lymphohistiocytosis; IM: infectious mononucleosis; PCR: polymerase chain reaction.

## Competing interests

The authors declare that they have no competing interests.

## Authors' contributions

YKJ carried out most of the studies and drafted the manuscript. GL and SY participated parts of the studies and writing. ZDX and KLS provided consultation and preparation of the final report. All authors read and approved the final manuscript.
